# A Glass–Ultra-Thin PDMS Film–Glass Microfluidic Device for Digital PCR Application Based on Flexible Mold Peel-Off Process

**DOI:** 10.3390/mi13101667

**Published:** 2022-10-04

**Authors:** Yanming Xia, Xianglong Chu, Caiming Zhao, Nanxin Wang, Juan Yu, Yufeng Jin, Lijun Sun, Shenglin Ma

**Affiliations:** 1 Department of Mechanical & Electrical Engineering, Xiamen University, Xiamen 361005, China; 2 Institute for Chemical and Bioengineering, ETH Zürich, 8093 Zürich, Switzerland; 3 School of Electronic and Computer Engineering, Peking University Shenzhen Graduate School, Shenzhen 518055, China; 4 School of Mechatronical Engineering, Beijing Institute of Technology, Beijing 100081, China

**Keywords:** digital polymerase chain reaction, flexible mold, glass–polydimethylsiloxane–glass, soft peel-off process, ultra-thin patterned polydimethylsiloxane film, water evaporation inhibition

## Abstract

The microfluidic device (MFD) with a glass–PDMS–glass (G-P-G) structure is of interest for a wide range of applications. However, G-P-G MFD fabrication with an ultra-thin PDMS film (especially thickness less than 200 μm) is still a big challenge because the ultra-thin PDMS film is easily deformed, curled, and damaged during demolding and transferring. This study aimed to report a thickness-controllable and low-cost fabrication process of the G-P-G MFD with an ultra-thin PDMS film based on a flexible mold peel-off process. A patterned photoresist layer was deposited on a polyethylene terephthalate (PET) film to fabricate a flexible mold that could be demolded softly to achieve a rigid structure of the glass–PDMS film. The thickness of ultra-thin patterned PDMS could reach less than 50 μm without damage to the PDMS film. The MFD showcased the excellent property of water evaporation inhibition (water loss < 10%) during PCR thermal cycling because of the ultra-thin PDMS film. Its low-cost fabrication process and excellent water evaporation inhibition present extremely high prospects for digital PCR application.

## 1. Introduction

The concept of digital polymerase chain reaction (dPCR) was first introduced by Vogelstein in 1999 [1]. By dispersing a sample into tens to tens of thousands of reaction units, each unit contains either a few or no DNA target molecules, which are analyzed statistically according to the fluorescence signal of each reaction unit after PCR thermal amplification [2,3,4]. Compared with conventional PCR, dPCR has the advantages of lower regent consumption, detection of more reaction units simultaneously, and not relying on calibration curves to quantify [5,6,7].

The problem of the easy evaporation of the sample in the reaction unit is a big challenge for the dPCR microfluidic device (MFD), which influences the accuracy and reliability of the dPCR results [8,9,10,11,12]. A sandwich structure of a microfluidic device, glass–PDMS–glass (G-P-G), can solve this problem. Compared with the traditional PDMS device, a cover glass is present on the top of the PDMS bulk, which can effectively suppress the sample loss in the reaction unit in the vertical direction during PCR thermal cycles [13,14,15,16,17,18]. However, the PDMS sidewall contacting the air leads to water losses through the porous PDMS layer to the outside. The rate of water losses is related to the area of the contact air, which is the thickness of the PDMS layer. Therefore, controlling the thickness of the middle PDMS layer is a critical issue. Tian proposed a G-P-G MFD with a vapor-proof layer (VPL) to moisturize or restrain evaporation caused by the gas permeability of PDMS under thermal cycling to solve the evaporation problem of the G-P-G MFD [19]. The water injected into the VPL in advance can effectively solve the evaporation problem. Still, the multi-layer structure makes its fabrication complex, and the liquid injection step needs to be used in combination with a pre-degassed PDMS pump.

Moreover, the ultra-thin PDMS layer in the G-P-G MFD is another effective method to inhibit water loss, but its fabrication is a significant challenge. The ultra-thin PDMS layer, with a thickness of less than 200 μm, is easily deformed, curled, or even damaged during the peel-off operation. Recently, many scholars have researched methods to solve the G-P-G MFD fabrication challenge. Inglis released a rigid PDMS film–glass from a rigid master with a blade, but it easily damaged the PDMS film and rigid master [20]. A flexible holder was used as a temporary tool to peel off a PDMS film to avoid damage to the rigid master. After peeling off the flexible holder and PDMS film from a rigid master, the rigid PDMS film–glass was bonded to a rigid glass cover and removed from the temporary holder [21]. The bonding strength between the holder and the PDMS film damaged the PDMS film. Liu reported a method to fabricate the G-P-G MFD using a 0.5 kg weight to extrude the excessive PDMS prepolymer [22]. This fabrication method had a limitation in that it could not control the thickness of the PDMS film precisely. Zhou proposed a caramel sacrificial mold to avoid peeling off between rigid materials [23]. The caramel sacrificial mold with complex removal steps and multiple molding processes makes its fabrication complex and inefficient. Moreover, all the aforementioned methods could not control the PDMS film thickness by pressing the PDMS prepolymer on the master. The methods based on a spin-coated PDMS layer to fabricate microfluidic channels by reactive ion etching (RIE) [24,25,26] and laser pyrolysis [27] were proposed to control the PDMS film thickness, both of which needed special and expensive equipment with low efficiency and high cost to fabricate.

Compared with the soft lithography PDMS fabrication process with demolding of the soft PDMS bulk from a rigid SU8-Si wafer mold [28], we presented a fabrication process with the demolding of a flexible patterned mold from a rigid PDMS film–glass cover for G-P-G MFD fabrication. A flexible patterned mold was developed and fabricated by one-step lithography on the polyethylene terephthalate (PET) film. The flexible patterned mold ensured that the rigid PDMS film–glass was not damaged or deformed after peeling off. This method proposed a simple manufacturing process and a low-cost and high-yield G-P-G microfluidic device fabrication process, which made the G-P-G microfluidic devices more accessible to researchers. Finally, a water loss inhibition experiment under PCR thermal conditions was presented to verify the excellent prospect in the dPCR application of the G-P-G MFD with the ultra-thin PDMS film.

## 2. Materials and Methods

### 2.1. Manufacturing Process Design

A flexible mold with patterned photoresist (PR) structures was fabricated by a lithography process on a PET film. A commercially available PET film (3M, Saint Paul, MN, USA) with a thickness of 100 μm was chosen to ensure flexibility and no easy deformation. The melting point of the PET film is about 260 °C [29,30], which is significantly higher than the baking and reflow temperatures of the typical PR. Because of its good chemical resistance, the PET film is not easily degraded or dissolved by typical organic solvents, including regular PR developers. Moreover, PET is an extremely low-cost material, 1000 times lower than a silicon wafer. The PR on the PET film was AZ 4620-positive PR (MicroChem, Round Rock, TX, USA), which could reflow to form a quake valve [31]. Moreover, the adhesion of AZ4620 PR on the PET film was much better than that of SU8 negative PR on the PET film.

**Figure 1:**
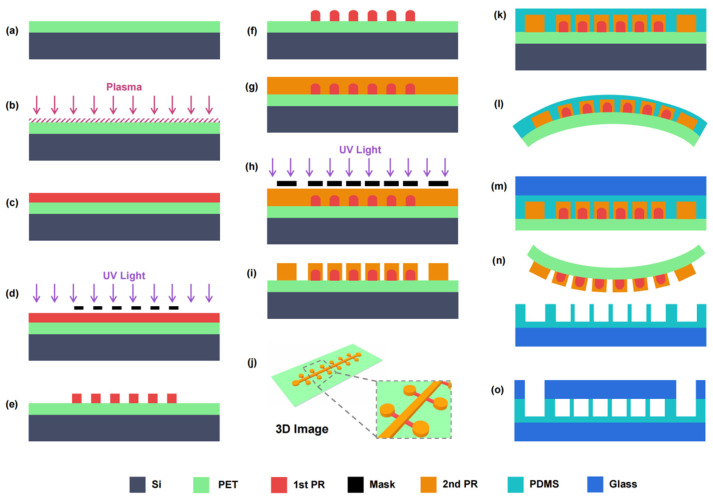
A fabrication process of the G-P-G MFD with an ultra-thin patterned PDMS film by a flexible soft mold peel-off process: (**a**) PET film attached to a 4-inch silicon wafer. (**b**) Oxygen plasma treatment on the PET surface. (**c**) First AZ 4620 PR layer with a height of 15 μm spin-coating. (**d**,**e**) Exposing and developing to form branch channel structures. (**f**) First AZ 4620 PR layer reflowing. (**g**) Second AZ 4620 PR layer with a height of 30 μm spin-coating. (**h**,**i**) Exposing and developing to form main channels and chamber structures. (**j**) A 3D image of the flexible mold. (**k**) A 10:1 mixture of PDMS spin-coating. (**l**) A flexible mold with PDMS film detaching from the rigid wafer. (**m**) Oxygen plasma bonding between PDMS surface and a cover glass. (**n**) Flexible mold peel-off from the PDMS film–glass structure. (**o**) Oxygen plasma alignment bonding between a rigid glass–PDMS film structure and a laser-drilled glass.

The fabrication process of a flexible mold is shown in Figure 1a–j. First, a PET film cut into a 4-inch round shape was attached to the surface of a reusable 4-inch Si wafer to ensure the fabrication process compatibility with the standard lithography process (Figure 1a). The PET surface was oxygen plasma-treated with an EMITECH K1000X plasma asher (Quorum Technologies, Wealden, UK) at 100 W for 60 s to improve the adhesion between AZ4620 PR and PET (Figure 1b). Then, a layer of AZ 4620 PR with a height of 15 μm was spin-coated on the treated PET surface, which required one spin-coating at 1500 rpm for 30 s with prebaking at 115 °C for 180 s (Figure 1c). The first layer of AZ 4620 PR was pattern-exposed using an MA6 mask aligner (SUSS, Munich, Germany) with 1400 mJ/cm^2^ (Figure 1d) and then developed into an AZ 326 MIF developer (MicroChem, Round Rock, TX, USA) for 4 min (Figure 1e). The wafer was baked on a hot plate at 160 °C for 2 h to change the photosensitivity of the first AZ 4620 PR layer so as to ensure that the first layer structure was not influenced by the second lithography (Figure 1f). Then, the second layer of AZ 4620 PR with a height of 30 μm was spin-coated on the first PR layer, which needed twice spin-coating at 1500 rpm for 30 s with prebaking at 110 °C for 80 s and 115 °C for 180 s, respectively (Figure 1g). The second PR layer was pattern-exposed with 2000 mJ/cm^2^ and developed for 10 min (Figure 1h,i). Finally, after hard baking at 110 °C for 120 s, a flexible PET mold with two patterned AZ 4620 PR structure layers of 15 μm and 30 μm in height was fabricated (Figure 1j).

Based on the PET flexible mold, the fabrication process of the G-P-G MFD is shown in Figure 1k–o. A 10:1 mixture of Sylgard 184 PDMS (Dow Corning, Midland, TX, USA) was spin-coated on the flexible mold at 1500 rpm for 30 s after silanization. A 50 μm-thick PDMS film with a patterned structure on the flexible mold was kept for 1 h and baked in an oven at 70 °C for 2 h to level and cure the PDMS layer (Figure 1k). The flexible mold with the PDMS layer was detached from the silicon wafer to remove the rigid substrate (Figure 1l). After washing with acetone and isopropyl alcohol, the silicon could be reused to save cost. The PDMS layer and a cover glass with 50 μm thickness were plasma bonded after oxygen plasma treatment at a power of 100 W for 60 s (Figure 1m). Then, the flexible mold was bent and peeled off softly from the rigid PDMS film–glass structure (Figure 1n). A laser-drilled glass with 100 μm thickness was aligned and bonded with the PDMS film after oxygen plasma treatment to fabricate a 200 μm-thick G-P-G MFD (Figure 1o). Optionally, a PDMS holder with through-holes was alignment bonded on the top of the cover glass for tubing.

### 2.2. Design of Digital PCR MFD

**Figure 2:**
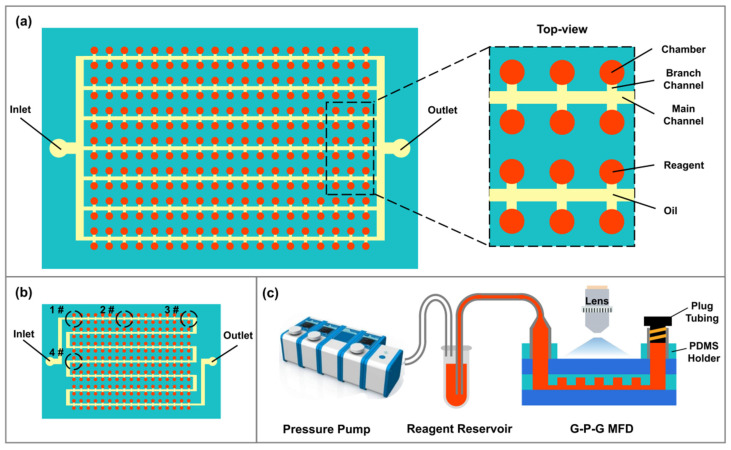
Schematic of the G-P-G MFD design with different main channel shapes: (**a**) parallel-shaped and (**b**) S-shaped, which is with different distances from I/O ports, the distance increase from 1# to 4#. (**c**) Schematic of the G-P-G MFD leakage test experiment platform setup. In the schematic, the red color is the reagent, and the yellow one is oil. The Aquamarine color is PDMS chip, and the blue one is glass cover.

A chamber cdPCR (cdPCR) MFD with 2160 digital microchambers, which could form the partitioned droplets, was designed to validate the G-P-G MFD dPCR application. Its inhibition ability of water evaporation was evaluated using the water volume loss rate in the partitioned chambers under PCR thermal cycling. Every microchamber was connected to the main channel with its branch channel. All the rooms were 30 μm in height and 150 μm in diameter. The branch channels were 15 μm in height and 40 μm in width. The main channels were 30 μm in height and 80 μm in width (Figure 2a). Besides, we designed a cdPCR G-P-G MFD with an S-shaped main channel compared with the parallel-shaped main channel to investigate the influence of the distance from the inlet and outlet on water evaporation (Figure 2b). A leakage test experiment platform was set up to test the leakage of the G-P-G MFD under the microfluidic operation condition. After the outlet was plugged, a Flow EZ pressure pump (Fluigent, Le Kremlin-Bicetre, France) was connected with a reagent reservoir to inject the liquid into the G-P-G MFD and observe it under a Ti-E inverted microscope (Nikon, Minato, Japan) (Figure 2c).

### 2.3. Experimental Setup and Evaluation of Evaporation Characteristics

**Figure 3:**
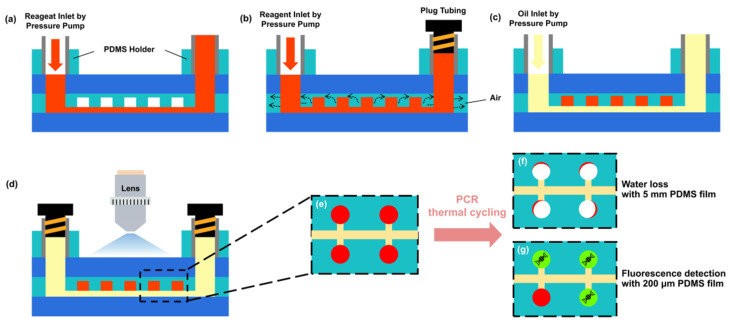
Operation procedure of the cdPCR G-P-G MFD: (**a**) filling the main channels with reagent using a pressure pump. (**b**) Plugging the outlet with a plugged tube and filling the chamber with the reagent. (**c**) Unplugging the tube in the outlet and injecting fluorinated oil to move out the reagent in the main channels to partite the water-phase reagent in the chambers. (**d**) After removing the injection tube, plugging inlet, and outlet. (**e**) Top view schematic of microchambers in cdPCR G-P-G MFD. (**f**) Serious water volume loss in the G-P-G MFD with a 5 mm-thick PDMS film after PCR thermal cycling. (**g**) Pretty low water volume loss in the G-P-G MFD with a 200 μm-thick PDMS film after PCR thermal cycling. The red color is the reagent, the yellow one is oil. The Aquamarine color is PDMS chip, and the blue one is glass cover. The white color is air after reagent evaporation, and the green one is fluorescence.

The operating procedure of the cdPCR G-P-G MFD is shown in Figure 3a–g. A water-phase simulation PCR regent (food dye) was injected into the MFD, and the main channels were filled with it using a pressure pump at 40 mBar pressure via a PDMS holder (Figure 3a). After a tubing was plugged in the outlet port to ensure that the liquid in the MFD did not flow out, the inlet pressure increased to 500 mBar. The water-phase reagent squeezed out the air in the chamber and then filled the entire chamber because of the gas permeability of PDMS (Figure 3b). After removing the plug tubing, an FC 3283 fluorinated oil (3M, Saint Paul, MN, USA) was injected into the main channels in the MFD with the pressure pump at 30 mBar. It pushed the water-phase reagent in the main and branch channels out of the MFD and replaced them to separate the water-phase regent in the chambers (Figure 3c). An inverted microscope was used to observe the water volume loss rate in the chamber under every PCR thermal cycling condition. After plugging the inlet and outlet ports, the cdPCR G-P-G MFD was transferred to a MasterCycler Nexus flat thermocycler (Eppendorf, Hamburg, Germany) to perform PCR thermal cycling (Figure 3d). According to the cdPCR amplification thermal condition [32,33,34], the simulation PCR thermal condition was set up in two steps. First was a “hot start” at 95 °C for 10 min to activate the Taq DNA polymerase, followed by 45 cycles of 95 °C for 20 s and 60 °C for 40 s to amplify the target DNA. The principle of the evaporation test is shown in Figure 3e–g. The G-P-G MFD with a 5 mm-thick PDMS film had a serious water volume loss (Figure 3f). In contrast, the G-P-G MFD with a 200 μm-thick PDMS film showcased a rather low water volume, whose fluorescence could be detected under a fluorescence microscope (Figure 3g).

## 3. Results and Discussion

### 3.1. Fabrication of Flexible Mold

Assembling a PDMS film with structures and rigid glass without damaging and deforming the PDMS film is a big challenge for G-P-G MFD fabrication because of the fragile PDMS film [35]. We proposed a G-P-G fabrication process involving the peel-off of a flexible mold, which replaced the flexible PDMS bulk peel-off of the soft lithography process. The flexible mold was softly demolded from a rigid PDMS film–glass structure to fabricate the G-P-G MFD efficiently and without damage.

**Figure 4:**
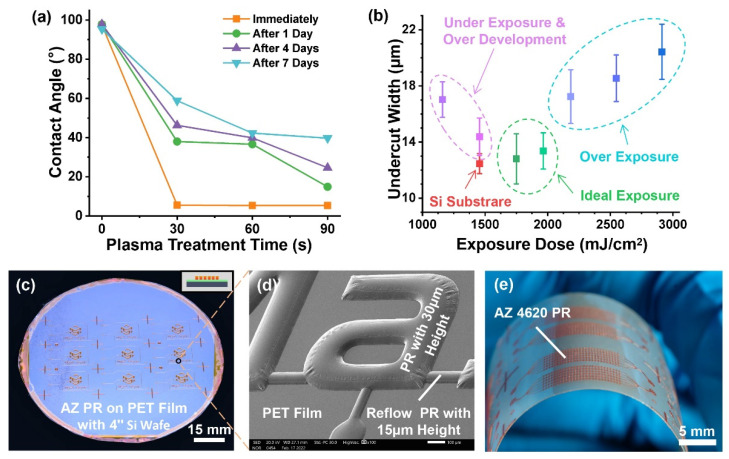
Fabrication results of the flexible mold: (**a**) influence on the contact angle of the PET surface with different oxygen plasma treatment time and standing time. (**b**) Effect of different exposure doses on single-side undercut width of the 20 μm-height PR structure. Two-layered PR structures with 5 μm-height reflow PR and 30 μm-height PR on the PET film: (**c**) 4-inch wafer picture and (**d**) SEM picture. (**e**) Excellent adhesion and flexibility of AZ 4620 PR on the PET film.

As the most critical technology for the fabrication process, the flexible mold with patterned PR structures was fabricated by lithography on a PET film surface. Compared with the wettability of the silicon wafer, PET wettability was much more hydrophobic, influencing the adhesion with PR. SU8 PR, the most used PR for soft lithography, could not be spin-coated directly on a PET surface. In contrast, AZ 4620 PR had a better adhesion with a PET surface and could be spin-coated directly. However, directly spin-coating on the PET surface could not achieve enough adhesion for PDMS mold demolding. The oxygen plasma treatment of the PET surface was a simple and effective method to alter the wettability [36], thereby improving the adhesion with PR. The wettability of the PET surface influenced by different oxygen plasma and standing times is shown in Figure 4a. The plasma treatment could significantly improve the PET surface hydrophilicity, lasting several days. More plasma treating time could extend the hydrophilicity time of the PET surface. After plasma treatment, AZ 4620 and SU8 PRs could spin coat on the PET surface well. However, the SU8 patterned structures could not attach to the bending PET film after plasma treatment, while the AZ 4620 patterned structures attached to the PET film excellently.

The PET surface reflection coefficient and the thickness between it and the silicon wafer, which was the holder to ensure compatibility with the MEMS process, influenced the exposure dose because of the optical transparency of a PET. A single-side undercut of a 20 μm-height PR structure under different exposure doses is shown in Figure 4b. The exposure dose on the PET film was significantly more compared with the exposure dose of the minimal undercut, which was the optimal width, on a bare silicon substrate. The underexposure PR dissolution needed much more development time, dissolving the unexposed area because of the extension of time. The reflection lights exposed the edges under the patterned mask structure when the exposure dose exceeded the optimal dose. The underexposure and over-exposure doses resulted in an increase in the undercut of the PR structure.

Due to the exposure area dissolution property of positive PR, it was difficult to fabricate a multi-layer structure similar to the negative PR, such as SU8 [37]. The chemical properties of AZ 4620 PR could be changed to not dissolve in the developer after exposure by reflow baking. The reflowing AZ 4620 PR could fabricate the multi-layer structure, and its curved top surface was used in the quake-valve application [38]. The reflowing temperature of AZ 4620 PR is 160 °C, which is lower than the melting point of PET, but higher than its glass transition temperature [39]. After baking at 160 °C for hours, the PR patterned structure on the PET film–silicon wafer was not deformed because of the softening PET at above glass transition temperature attached to the rigid silicon wafer. Figure 4c,d shows the whole wafer picture and SEM picture of two-layered PR structures with 15 μm-height reflow PR and 30 μm-height PR on the PET film–silicon wafer. The AZ 4620 PR multi-layer structure was formatted uniformly on the PET film with solid adhesion (Figure 4e), which was stable for at least five replica moldings.

### 3.2. Fabrication of G-P-G MFD

**Figure 5:**
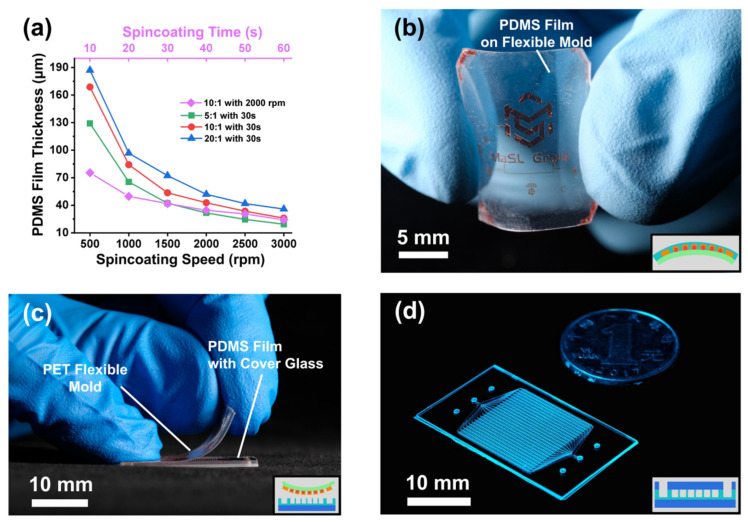
Fabrication results of G-P-G MFD: (**a**) data of the PDMS thickness under different spin-coating parameters and PDMS mixture ratios. (**b**) Flexible patterned mold with the PDMS film. (**c**) Process of PET flexible mold peeling off from the PDMS film with a glass cover. (**d**) G-P-G MFD with a total thickness of 200 μm.

The G-P-G MFD thickness could be thinner and more precisely controllable by the PDMS spin-coating process, which has a massive prospect for thickness-sensitive applications. Since the AZ4620 PR-PET surface was different from the SU8-Si surface, the PDMS thickness on the PET film with different ratios, rotation speeds, and rotation times was developed (Figure 5a). The PDMS film thickness ranged from 19.4 to 187.1 μm, which could fit mostly ultra-thin G-P-G MFD. After spin-coating and curing, the PDMS film was still attached to the rigid structure of the silicon wafer. Removing the silicon wafer substrate was necessary to form a flexible PDMS-PET film structure before PDMS–glass bonding so as to avoid the peel-off process among two rigid substrates. A flexible PDMS film with PET mold showed an excellent bending property (Figure 5b). The rigid PDMS film–glass structure was formatted by plasma bonding with a cover glass and then peeling off the flexible mold. After plasma bonding with a cover glass, a rigid PDMS film–glass structure was formatted by peeling off the flexible mold (Figure 5c), which needed a porous ceramic chuck table to fix the cover glass when the glass thickness was less than 100 μm. A G-P-G MFD with a total thickness of 200 μm was fabricated and is shown in Figure 5d.

### 3.3. Sample Filling and Self-Partition

We designed a pressure pump injection filling method for the cdPCR G-P-G MFD, which was more straightforward with a more friendly operating condition than the methods needing a vacuum condition [40,41,42]. In this method, a reagent was injected into the MFD using a pressure pump with a certain pressure after a plug blocked the MFD outlet port to ensure that the liquid could not flow out of the MFD. The reagent with positive pressure pushed out the air in a chamber and filled the chamber because of the air permeability of PDMS. When pushing out air with a certain pressure, it was crucial to ensure the bonding strength and tightness of the inlet and outlet ports of the G-P-G MFD. The G-P-G MFD with the PDMS holder was verified to withstand pressures above 2 Bar without any leakage using the leakage test experiment platform in Figure 2c. In addition, a thermal stability experiment was set up and verified that the channel deformation was only 0.7% in the 200 μm-diameter chamber of the G-P-G MFD with a 200 μm-thick PDMS film under the temperature variation from 20 °C (room temperature) to 200 °C. The difference in CTE between the borosilicate glass (3.3 × 10^−6^/°C) [43] and PDMS (291 × 10^−6^/°C) [44] little influenced the G-P-G MFD.

**Figure 6:**
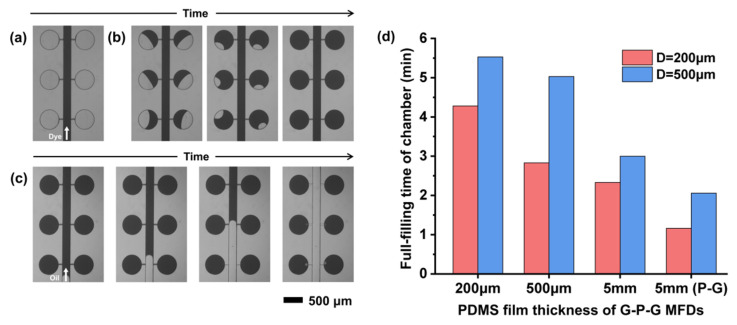
Sample filling and self-partition: (**a**) the food dye injection at 40 mBar. (**b**) Filling the chambers with the food dye at 500 mBar. (**c**) Process of the fluorinated oil pushing the excess reagent out of the main channels. (**d**) Filling time of chambers in the G-P-G MFD with different diameters and PDMS thicknesses.

After filling the chambers with the reagent, a fluorinated oil was injected into the channels to partite the reagent in the chambers and form the droplets. A red food dye solution is simulated as the PCR regent for filling and partitioning to visually demonstrate the functionality of the cdPCR MFD. First, the food dye was injected into the MFD with a low pressure (40 mBar) to fill the main channel when the outlet port was not blocked (Figure 6a). The chambers could not be filled by pushing out the air now because of no pressure difference between the chamber and the outside world. After blocking the outlet port and increasing the injection pressure to 500 mBar, the pressure in the MFD was much higher than the outside pressure because the liquid could not flow out. The fluid with higher pressure compressed the air in the chamber, resulting in higher compressing air pressure than that outside. The air was discharged from the chamber through the PDMS film, and the process is shown in Figure 6b. Removing the plug tubing ensured that the liquid could flow out of the MFD again. A fluorinated oil was injected into the MFD and pushed the excess reagent in the channels out of the device, which separated the reagent in the chambers to form droplets (Figure 6c). A larger number of independent droplets, the base of the dPCR MFD, could be automatically created without any valve or external device. Figure 6d shows the filling time of different chamber diameters in the MFDs with different PDMS thicknesses under 500 mBar injection pressure. For the G-P-G MFD, the reagent filling speed was significantly related to the PDMS thickness due to the existence of a glass cover on the top of the PDMS film. The filling speed decreased significantly with the decrease in the PDMS thickness. The decreasing PDMS thickness resulted in a decrease in the area of the PDMS sidewall used for air discharge, thereby reducing the air discharge speed. The filling time increased from 3 min to 5.5 min for a chamber with a 500 μm diameter with the PDMS thickness decreasing from 5 mm to 200 μm. Even when the time increased significantly, the total operation time was still lower than that for the reported vacuum chamber methods [45,46,47]. Moreover, the whole operation process of this method could be achieved under normal pressure, which made the operation process easier.

### 3.4. Evaluation of Evaporation of the G-P-G MFD

Water evaporation loss is a severe challenge for the dPCR system, which may increase the concentration of PCR reagent and thereby inhibit PCR. A glass cover attached to the top of PDMS MFD to form a G-P-G sandwich MFD is an effective solution. Compared with previously reported barrier methods such as parylene coating [48,49] and polymer embedding [50,51], the glass cover has much lower vapor permeability, about four orders less than propylene. However, still some water evaporation loss occurs in the G-P-G MFD, especially in the periphery area near the PDMS sidewall, under the PCR thermal cycles [6]. According to Fick’s law of diffusion, the amount of water vapor is proportional to the diffusion area, which is the PDMS sidewall area of the G-P-G MFD contacting outside. Therefore, the thickness of the PDMS film is significantly related to the water evaporation in the G-P-G MFD. We proposed a G-P-G MFD of an ultra-thin PDMS film to inhibit water evaporation by reducing the PDMS thickness. The inhibitory effect of the PDMS thickness, especially the ultra-thin PDMS film, on evaporation under the PCR thermal condition was investigated to demonstrate the performance and validity of the G-P-G MFD in reducing water loss.

**Figure 7:**
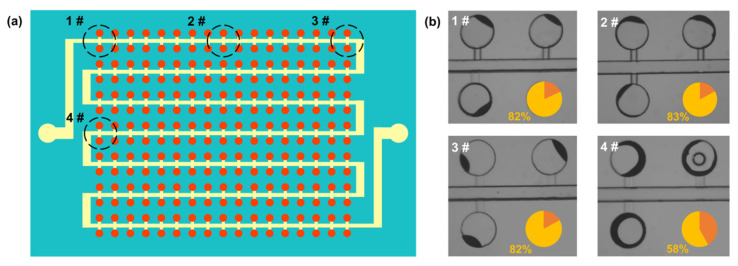
(**a**) Schematic of different evaluated locations in the cdPCR MFD with an S-shape main channel, the red color is the reagent, the yellow one is oil, and the Aquamarine color is PDMS chip. (**b**) Water loss percentage in the chambers at different distances from the ports and PDMS sidewall.

An S-shape main channel cdPCR G-P-G MFD with 5 mm-thick PDMS bulk was designed and investigated to evaluate the influence of distance from the inlet and outlet ports on the evaporation inhibition, as shown in Figure 7a. The water loss of areas in the chambers at different distances from the ports and PDMS sidewall is shown in Figure 7b. The water loss of areas at 1#, 2#, and 3# was almost the same, implying that the water loss effect in the periphery area was unrelated to the distance from the inlet and outlet ports. However, the water loss in areas at 1# and 4# was not the same, verifying that the water loss was related to the distance from the PDMS sidewall.

**Figure 8:**
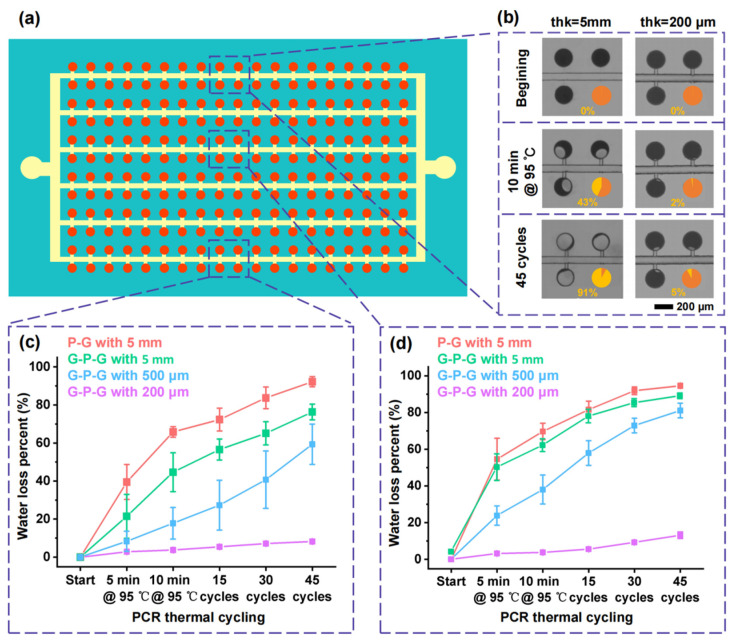
Evaporation result of the central and peripheral chambers in cdPCR MFD: (**a**) location schematic of the central and peripheral chambers in the cdPCR MFD, the red color is the reagent, the yellow one is oil, and the Aquamarine color is PDMS chip. (**b**) Water loss process in the peripheral chambers under PCR thermal cycling of G-P-G MFDs with 5 mm and 200 μm-thick PDMS films. Water loss percentage in the chambers with PCR thermal cycling in the G-P-G MFD with different PDMS film thicknesses: (**c**) central chambers and (**d**) peripheral chambers.

The variations in the food dye loss percentage in the chambers with the change in the number of thermal cycles were investigated to quantify the effect of the PDMS thickness in the G-P-G MFD on evaporation inhibition. Figure 8 shows the food dye loss in the central and peripheral chambers of cdPCR MFDs with the different thicknesses of the PDMS film under PCR thermal cycling, and the location schematic of the chambers in the cdPCR MFD is shown in Figure 8a. For the G-P-G MFD with 5 mm thickness of the PDMS bulk, the food dye loss percentage in the chamber increased significantly with the progress of thermal cycling. In contrast, the food dye loss percentage in the chamber of the G-P-G MFD with 200 μm thickness increased little, as shown in Figure 8b. The food dye loss percentage in the chamber increased with the increase in the thickness of the PDMS film in G-P-G MFDs, and the difference was more significant as the number of PCR thermal cycling increased, as shown in Figure 8c. The reason was that the PDMS film thickness determined the sidewall area connecting the air, which could influence the water loss speed. Moreover, the water evaporation inhibition of the glass cover on the top of the PDMS was verified by comparing it with the effect of a 5 mm-thick PDMS bulk in the G-P-G and PDMS–glass MFD. Compared with the ones in the central chambers, the food dye loss percent in the peripheral chambers was much more because the smaller distance from the PDMS sidewall improved the evaporation speed. For the G-P-G MFD with a 500 μm-thick PDMS film, the food dye loss percentage in the central chambers was about 50%, but the ones in the peripheral chambers increased to almost 80%, which seriously influenced the dPCR detection, which is shown in Figure 8d. However, when the thickness was reduced to 200 μm, the food dye loss percentage in the peripheral chambers decreased significantly. The thicknesses in the central and peripheral chambers were less than 10% after the PCR thermal condition, which did not influence the dPCR detection. It demonstrated that the proposed G-P-G MFD of the ultra-thin PDMS film (thickness less than 200 μm) had an extremely low water loss percentage under the PCR thermal condition, especially in the peripheral chambers, due to its ultra-thin PDMS film. The G-P-G MFD of the ultra-thin PDMS film presents excellent application prospects in digital PCR and other high-temperature microfluidic biochemical reactors.

## 4. Conclusions

This study demonstrated a low-cost facile process to fabricate a G-P-G MFD based on a flexible mold peel-off. A PET flexible mold with multi-level microstructures was fabricated by researching the lithography of AZ 4620-positive PR on an ultra-low-cost PET film. A low-cost, thickness-controllable, high-fabrication yield G-P-G MFD fabrication process was developed based on the PET flexible mold, which greatly improved the feasibility of G-P-G MFD fabrication. A straightforward operation cdPCR microfluidic platform was demonstrated using the G-P-G MFD with the ultra-thin PDMS film. Moreover, the evaporation inhibition of the G-P-G MFD with the PDMS films of different thicknesses was investigated by quantifying the water loss percentage in the chambers under PCR thermal cycling. The G-P-G MFD with the ultra-thin PDMS film (<200 μm) showed excellent water evaporation inhibition, which is highly promising for digital PCR applications.

## Data Availability

The data presented in this study are available on request from the corresponding author.
